# Cardiac abnormalities and repolarization variability in epilepsy: influence of antiseizure medications and treatment response

**DOI:** 10.3389/fcvm.2025.1617114

**Published:** 2025-10-16

**Authors:** Cagdas Kaynak, Ayfer Ertekin, Mehmet Attila Erkuzu

**Affiliations:** ^1^ Depatment of Cardiology, Siirt Education and Research Hospital, Siirt, Türkiye; ^2^ Department of Neurology, Siirt Education and Research Hospital, Siirt, Türkiye

**Keywords:** epilepsy, anticonvulsants, atrial enlargement, cardiac remodeling, electrocardiography

## Abstract

**Objective:**

This study aimed to investigate the effects of different antiseizure medications (ASMs) and clinical treatment response on interictal electrocardiographic (ECG) repolarization indices and echocardiographic structural and functional cardiac parameters in patients with epilepsy.

**Methods:**

This single-center, prospective, cross-sectional study was conducted between August 2024 and February 2025 at the Neurology and Cardiology Departments of Siirt Training and Research Hospital. The study included 97 patients with epilepsy and 57 age- and sex-matched healthy controls. Patients were classified as drug-sensitive or drug-resistant based on the International League Against Epilepsy (ILAE) criteria. While both monotherapy and polytherapy users were included in clinical comparisons, subgroup analyses by ASM type involved only monotherapy patients. ECG and echocardiographic parameters were assessed. Group comparisons and correlation analyses were performed using analysis of variance (ANOVA), chi-square test, and Pearson correlation.

**Results:**

No significant differences in demographic or interictal ECG repolarization indices were found between the drug-sensitive, drug-resistant, and control groups (*P* > 0.05). Both epilepsy groups had significantly higher left atrial volum index (LAVi) compared to controls (*P* < 0.001), while A velocity and lateral E′ were significantly lower. Carbamazepine (CBZ) group showed the highest LAVi, though without correlation to treatment duration. In contrast, valproic acid (VPA) and levetiracetam (LEV) groups exhibited significant positive correlations between treatment duration and LAVi (r = 0.776, *P* = 0.002; r = 0.571, *P* = 0.002, respectively).

**Conclusion:**

Treatment with ASMs in epilepsy is associated with left atrial enlargement, particularly with CBZ. The absence of interictal ECG repolarization differences suggests that isolated assessments may be insufficient, supporting long-term cardiac monitoring in epilepsy management.

## 1 Introduction

In patients with epilepsy, catecholaminergic surges and hypoxic episodes associated with seizures may, over time, lead to structural cardiac damage, impairing both the mechanical and electrical functions of the heart (1). Previous studies have shown alterations in electrocardiographic (ECG) parameters such as QT interval (QTint), corrected QT (QTc), and Tpeak-Tend interval (Tp-e) interval in patients with epilepsy, which may contribute to an increased risk of sudden cardiac death (SCD) and sudden unexpected death in epilepsy (SUDEP) (2). In addition to electrophysiological abnormalities, several ECG studies have reported structural and functional cardiac changes in this population, including reduced left ventricular ejection fraction (LVEF), increased myocardial stiffness, elevated filling pressures, and enlargement of the left atrium (LA) (3, 4). However, it is important to note that repolarization abnormalities on ECG are typically observed during ictal and postictal periods (5, 6). Evaluating these parameters during the interictal period may provide insight into the long-term subclinical effects of epilepsy on cardiac structure and function, thus offering greater value in risk stratification.

Antiseizure medications (ASMs)—particularly enzyme-inducing agents—have been associated with the development of structural and functional cardiac abnormalities and a predisposition to arrhythmias (7, 8). On the other hand, some ASMs have demonstrated a neutral cardiac profile even after long-term use (9). Cardiac involvement may also be more pronounced in patients with drug-resistant epilepsy, where autonomic dysfunction and persistent repolarization abnormalities have been more frequently reported (1, 10). Therefore, evaluating interictal ECG and echocardiographic parameters according to clinical response and ASM type may help identify long-term cardiac consequences more accurately.

In light of this evidence, the present study aimed to evaluate the effects of ASMs and treatment response on ECG repolarization indices, cardiac structural-functional parameters during the interictal period in patients with epilepsy.

## 2 Material and methods

### 2.1 Study participants

This single-center, prospective, cross-sectional observational study was conducted between August 2024 and February 2025 at the Neurology and Cardiology Departments of Siirt Training and Research Hospital. The study included 97 patients with epilepsy without any known history of cardiovascular disease and 57 age- and sex-matched healthy individuals as the control group. The diagnosis of epilepsy was established by a neurologist based on clinical evaluation and seizure history, in accordance with the International League Against Epilepsy (ILAE) guidelines, requiring at least two unprovoked seizures occurring more than 24 h apart.

Epilepsy patients were classified as having drug-resistant or drug-sensitive epilepsy based on their clinical response. Drug-resistant epilepsy was defined as the failure of adequate trials of two ASM regimens (monotherapy or combination) that were appropriately selected and tolerated, yet failed to achieve sustained seizure freedom. Drug-sensitive epilepsy was defined as seizure freedom for at least 12 months or three times the longest interseizure interval prior to treatment under the current ASM regimen (11).

Patients with known structural heart disease, arrhythmias, valvular disease, moderate-to-severe hypertension (HT), diabetes mellitus (DM), renal or hepatic failure, thyroid dysfunction, active infection, age under 18 years, symptomatic epilepsy, abnormal brain magnetic resonance imaging (MRI), irregular ASM use, or insufficient clinical follow-up were excluded.

During data collection, six patients were excluded from the study due to the presence of cardiac abnormalities: three with mitral valve prolapse, two with rheumatic mitral valve disease, and one with atrial fibrillation (AF). All ECG and transthoracic echocardiography (TTE) measurements were performed and interpreted by an experienced cardiologist who was blinded to the clinical grouping.

Both drug-sensitive and drug-resistant groups included patients receiving either monotherapy or polytherapy. However, to avoid potential drug interaction effects, subgroup analyses based on ASM type included only patients who were on monotherapy with sodium valproate (VPA) (*n* = 13), carbamazepine (CBZ) (*n* = 15), or levetiracetam (LEV) (*n* = 27), had at least one year of clinical follow-up, biannual electroencephalography (EEG) records, and therapeutic drug serum levels within reference ranges.

### 2.2 Electrocardiographic assessment

Standard 12-lead ECG recordings were obtained using a NIHON Kohden Cardiofax ECG device. The following parameters were measured:

• Basic rhythm parameters: heart rate (beats per minute), P-wave duration (Pwd, ms), PR interval (PRint, ms), and PR segment duration (PRsegd, ms).
• Depolarization and repolarization indices: QRS duration (QRSdur, ms), QTint (ms), QTc (ms), and T-wave duration (Twd, ms).
• Repolarization dispersion indices: Tp-e (ms), Tp-e/QT and Tp-e/QTc ratios (dimensionless) and frontal QRS-T angle [f(QRS-T), degrees].

All ECGs were recorded during the interictal period and analyzed by an experienced cardiologist blinded to the clinical and treatment grouping.

### 2.3 Echocardiographic assessment

In addition, all participants underwent transthoracic echocardiographic (TTE) evaluation using a Philips Affiniti CVx ultrasound system. The following structural and functional parameters were assessed:

• Left atrial measurements: anteroposterior (LA-AP), superoinferior (LA-SI), and mediolateral (LA-ML) diameters (cm), left atrial volume (LAV, mL), and left atrial volume index (LAVi, mL/m²).
• Left ventricular systolic function: EF (%), fractional shortening (FS, %), left ventricular end-diastolic diameter (LVEDD, cm), left ventricular end-systolic diameter (LVESD, cm).
• Left ventricular wall thickness and mass: Interventricular septal thickness (IVS, cm), left ventricular posterior wall thickness (LVPWT, cm), left ventricular mass (LVM, g), left ventricular mass index (LVMI, g/m²), and relative wall thickness (RWT).
• Diastolic function indices: Mitral E and A wave velocities (E, A; cm/s), E/A ratio, lateral mitral annular E′ velocity (Lateral E′, cm/s), E/E′ ratio, deceleration time of the E wave (DT, ms), and isovolumic relaxation time (IVRT, ms).

All echocardiographic measurements were performed in accordance with the the American Society of Echocardiography and the European Association of Cardiovascular Imaging guidelines by a cardiologist blinded to the clinical grouping (12).

### 2.4 Statistical analysis

All statistical analyses were performed using SPSS version 26.0 (IBM Corp., Armonk, NY, USA). The normality of distribution for continuous variables was assessed using the Kolmogorov–Smirnov test. Variables with normal distribution were expressed as mean ± standard deviation (SD). Categorical variables were expressed as percentages.

Comparisons of demographic characteristics, blood pressure values, ECG parameters (e.g., heart rate, Pwd, PRint, QT, QTc, Tp-e, Tp-e/QT, and Tp-e/QTc ratios), and echocardiographic structural and functional variables (e.g., LAV, LAVi, E/A, E/E’, and LVPWT) were initially conducted between three groups: drug-sensitive epilepsy, drug-resistant epilepsy and healthy controls. The same parameters were also compared among subgroups based on different monotherapy ASMs and the control group.

For continuous variables, analysis of variance (ANOVA) was used for group comparisons. *post-hoc* analyses were conducted using the Tukey-B test for variables with statistically significant differences. For the comparison of categorical variables the Pearson chi-square test was applied. Finally, among the monotherapy subgroups, the relationship between treatment duration and LAVi was assessed using Pearson correlation analysis, and the results were visualized graphically. A *p*-value < 0.05 was considered statistically significant in all analyses.

## 3 Results

### 3.1 Comparison of demographic and ECG parameters between drug-sensitive, drug-resistant, and control groups

The comparison of the participants’ ECG and demographic characteristics were presented in Table 1. No statistically significant differences were observed among the drug-sensitive, drug-resistant and control groups in terms of age, male gender ratio, blood pressure values, body mass index (BMI), body surface area (BSA) and ECG findings; including heart rate, Pwd, PRint, PRsegd, QRSd, Twd, QT, QTc, Tp-e, Tp-e/QT ratio, Tp-e/QTc ratio, and f(QRS-T) angle (*P* > 0.05). However, the treatment duration was significantly longer in the drug-resistant epilepsy group compared to the drug-sensitive group (*P* < 0.001).

**Table 1:** Comparison of electrocardiographic and demographic characteristics among drug-sensitive, drug-resistant epilepsy and control groups.

Variables	Control (*n* = 57)	Drug-sensitive epilepsy (*n* = 73)	Drug-resistant epilepsy (*n* = 24)	Total (*n* = 154)	*P-*value
Age (years)	33.98 ± 11.48	32.35 ± 13.16	34.08 ± 13.67	33.22 ± 12.59	0.72
Male gender (*n*, %)	21 (36.8)	42 (57.5)	13 (54.2)	76 (49.4)	0.057
Treatment duration (years)	–	9.98 ± 7.63	16.95 ± 8.62	11.71 ± 8.41	<0.001
SBP (mmHg)	113.15 ± 13.77	111.08 ± 18.14	112 ± 13.63	111.99 ± 15.91	0.764
DBP (mmHg)	68.94 ± 9.94	70.52 ± 9.84	71.12 ± 9.91	70.03 ± 9.86	0.562
BSA (m^2^)	1.77 ± 0.20	1.76 ± 0.22	1.80 ± 0.25	1.77 ± 0.22	0.671
BMI (kg/m^2^)	26.13 ± 4.79	24.94 ± 5.59	25.93 ± 5.56	25.53 ± 5.30	0.415
HR (bpm)	80.68 ± 13.45	79.31 ± 12.40	75.08 ± 14.80	79.16 ± 13.23	0.219
Pdur (ms)	76.57 ± 13.98	77.30 ± 16.04	80 ± 19.56	77.45 ± 15.86	0.674
PRint (ms)	148.87 ± 18.84	146.23 ± 18.91	150.91 ± 18.43	147.94 ± 18.77	0.512
PRsegdur (ms)	72.29 ± 19.24	68.94 ± 22.36	70.91 ± 20.90	70.49 ± 20.95	0.663
QRSdur (ms)	89.68 ± 10.94	89.42 ± 10.17	87.20 ± 9.84	89.17 ± 10.38	0.597
Twd (ms)	166.54 ± 33.87	165.61 ± 32.48	171.66 ± 43.97	166.90 ± 34.81	0.76
QT (ms)	358.66 ± 25.29	357.69 ± 24.58	364.25 ± 27.02	359.07 ± 25.16	0.539
QTc (ms)	393.28 ± 16.93	389.09 ± 17.67	388.41 ± 14.77	390.53 ± 17.00	0.306
Tp-e (ms)	66.75 ± 13.14	69.01 ± 14.26	73.12 ± 13.49	68.81 ± 13.81	0.164
Tp-e/QT	0.185 ± 0.034	0.192 ± 0.038	0.2 ± 0.032	0.191 ± 0.036	0.245
Tp-e/QTc	0.169 ± 0.032	0.177 ± 0.036	0.187 ± 0.032	0.175 ± 0.034	0.09
F(QRS-T)angle (degrees)	26.63 ± 18.65	21.47 ± 16.94	17.70 ± 13.16	22.79 ± 17.28	0.07

Data are presented as mean ± standard deviation for continuous variables and as number (percentage) for categorical variables. Group comparisons were performed using one-way ANOVA for continuous variables and Pearson's chi-square test for categorical variables. SBP, systolic blood pressure; DBP, diastolic blood pressure; BSA, body surface area; BMI, body mass index; HR, heart rate; Pdur, P-wave duration; PRint, PR interval; PRsegdur, PR segment duration; QRSdur, QRS duration; Twd, T-wave duration; QT, QT interval; QTc, corrected QT interval; Tp-e, Tpeak-Tend interval; Tp-e/QT, Tp-e to QT ratio; Tp-e/QTc, Tp-e to QTc ratio; F(QRS-T)angle, frontal QRS-T angle.

### 3.2 Comparison of echocardiographic systolic, diastolic, and left atrial measurements among groups

The findings regarding the comparison of the participants’ echocardiographic structural characteristics and diastolic and systolic function parameters were presented in Table 2. A velocity was significantly lower in both the drug-sensitive and drug-resistant epilepsy groups compared to the control group (*P* = 0.014, *P* = 0.006, respectively). Similarly, the lateral E velocity was significantly lower in the drug-sensitive and drug-resistant epilepsy groups than in the control group (*P* = 0.045, *P* = 0.023, respectively). The left atrium mediolateral diameter (LAMLD) was significantly lower in the drug-resistant epilepsy group compared to the control group (*P* = 0.005). LAV and LAVi were significantly higher in both the drug-sensitive and drug-resistant epilepsy groups compared to the control group (*P* < 0.001, *P* < 0.001, *P* < 0.001, *P* < 0.001, respectively). Additionally, LVPWT was significantly lower in the drug-sensitive epilepsy group than in the control group (*P* = 0.023).

**Table 2:** Echocardiographic comparison of systolic, diastolic, and left atrial measurements among drug-sensitive, drug-resistant epilepsy and control groups.

Variables	Control (*n* = 57)	Drug-sensitive epilepsy (*n* = 73)	Drug-resistant epilepsy (*n* = 24)	Total (*n* = 154)	*P-*value
EF (%)	63.12 ± 2.26	62.76 ± 2.44	62.12 ± 2.49	62.79 ± 2.39	0.228
E/A	1.36 ± 0.40	1.49 ± 0.53	1.45 ± 0.38	1.44 ± 0.46	0.325
E (cm/s)	95.81 ± 20.03	91.65 ± 20.70	86.35 ± 15.85	92.36 ± 19.9	0.137
A (cm/s)	75.17 ± 20.3	65.88 ± 17.68	61.07 ± 14.40	68.57 ± 18.94	0.002
E DT (ms)	160.43 ± 46.94	164.28 ± 45.38	176.54 ± 42.25	164.77 ± 45.53	0.347
Lateral E’ (cm/s)	76.32 ± 19.18	69.10 ± 15.35	65.43 ± 13.11	71.20 ± 16.98	0.01
IVRT (ms)	77.98 ± 12.11	79.21 ± 11.32	81.5 ± 11.11	79.11 ± 11.57	0.459
FS (%)	46.23 ± 4.30	45.33 ± 5.48	43.60 ± 5.60	45.39 ± 5.14	0.108
E/E'	9.23 ± 2.44	8.29 ± 2.93	9.84 ± 4.65	8.88 ± 3.13	0.063
LA-AP (cm)	4.90 ± 0.44	4.81 ± 0.58	4.72 ± 0.59	4.83 ± 0.54	0.374
LA-SI (cm)	3.84 ± 0.43	3.72 ± 0.46	3.71 ± 0.46	3.76 ± 0.45	0.305
LA-ML (cm)	3.46 ± 0.35	3.26 ± 0.54	3.09 ± 0.53	3.31 ± 0.49	0.003
LAV (ml)	40.91 ± 7.97	64.35 ± 17.4	64.01 ± 18.32	55.62 ± 18.59	<0.001
LAVi (ml/m^2^)	23.63 ± 4.65	37.10 ± 10.10	36.78 ± 10.62	32.07 ± 10.72	<0.001
LVEDD (cm)	4.41 ± 0.27	4.34 ± 0.26	4.35 ± 0.23	4.37 ± 0.26	0.238
LVESD (cm)	2.36 ± 0.21	2.36 ± 0.24	2.42 ± 0.2	2.37 ± 0.23	0.439
LVPWT (cm)	0.97 ± 0.13	0.90 ± 0.11	0.91 ± 0.1	0.93 ± 0.13	0.024
IVS (cm)	0.94 ± 0.13	0.91 ± 0.12	0.92 ± 0.14	0.92 ± 0.13	0.38
LVMI (g/m^2^)	79.92 ± 15.6	73.47 ± 14.58	74.25 ± 18.73	75.98 ± 15.86	0.059
LVM (g)	141.85 ± 33.43	129.54 ± 30.55	131.86 ± 35.70	134.46 ± 32.75	0.095
RWT	0.43 ± 0.06	0.41 ± 0.05	0.41 ± 0.06	0.42 ± 0.05	0.339

All variables are presented as mean ± standard deviation. Group comparisons were conducted using one-way ANOVA. *post-hoc* analyses for pairwise comparisons were performed using the Tukey-B test. EF, ejection fraction; E, early mitral inflow velocity; A, late mitral inflow velocity; E/A, E to A ratio; E DT, E-wave deceleration time; Lateral E′, lateral mitral annular E′ velocity; IVRT, isovolumic relaxation time; FS, fractional shortening; E/E′, E to E′ ratio; LA-AP, left atrial anteroposterior diameter; LA-SI, left atrial superoinferior diameter; LA-ML, left atrial mediolateral diameter; LAV, left atrial volume; LAVi, left atrial volume index; LVEDD, left ventricular end-diastolic diameter; LVESD, left ventricular end-systolic diameter; LVPWT, left ventricular posterior wall thickness; IVS, interventricular septum thickness; LVMI, left ventricular mass index; LVM, left ventricular mass; RWT, relative wall thickness.

### 3.3 Demographic and ECG characteristics by monotherapy AED type

The comparison of the ECG and demographic characteristics of the participants and the antiepileptic drugs used in monotherapy were presented in Table 3. No statistically significant differences were observed among the groups of CBZ, VPA, LEV and control group in terms of age, treatment duration, blood pressure values, body measurements (BMI, BSA), and ECG findings, including heart rate, Pwd, PRint, PRsegd, QRSd, Twd, QT, QTc, Tp-e, Tp-e/QT ratio, Tp-e/QTc ratio, and f(QRS-T) angle (*P* > 0.05). However, the male gender ratio was significantly higher in the VPA group compared to the control group (*P* = 0.007).

**Table 3:** Comparison of demographic and electrocardiographic parameters among ASM groups and control group.

Variables	Control (*n* = 57)	CBZ group (*n* = 15)	VPA group (*n* = 13)	LEV group (*n* = 27)	Total (*n* = 112)	*P*
Age (years)	33.98 ± 11.48	36.13 ± 13.01	29.69 ± 9.04	32.51 ± 12.58	33.41 ± 11.69	0.495
Male gender (*n*, %)	21 (36.8)	10 (66.7)	11 (84.6)	12 (44.4)	54 (48.2)	0.007
Treatment duration (years)	–	13 ± 8.29	11.23 ± 9.59	7.55 ± 6.58	9.15 ± 7.78	0.086
SBP (mmHg)	113.15 ± 13.77	116 ± 21.97	110.76 ± 13.20	109.62 ± 15.56	112.41 ± 15.37	0.582
DBP (mmHg)	68.94 ± 9.94	73.33 ± 11.12	67.69 ± 8.32	70.74 ± 9.97	69.82 ± 9.95	0.376
BSA (m^2^)	1.77 ± 0.20	1.83 ± 0.23	1.83 ± 0.23	1.74 ± 0.19	1.78 ± 0.20	0.433
BMI (kg/m^2^)	26.13 ± 4.79	25.33 ± 4.76	26.36 ± 7.32	25.18 ± 5.03	25.82 ± 5.14	0.829
HR (bpm)	80.68 ± 13.45	77.4 ± 12.30	81.46 ± 11.89	77.96 ± 12.43	79.67 ± 12.81	0.674
Pwd (ms)	76.57 ± 13.98	81.66 ± 14.59	77.92 ± 17.44	77.40 ± 14.76	77.61 ± 14.57	0.697
PRint (ms)	148.87 ± 18.84	151.2 ± 12.77	146 ± 19.30	146.14 ± 19.89	148.19 ± 18.32	0.804
PRsegdur (ms)	72.29 ± 19.24	69.53 ± 19.6	68.07 ± 14.18	68.74 ± 25.31	70.58 ± 20.26	0.836
QRSdur (ms)	89.68 ± 10.94	92.26 ± 9.06	86.92 ± 8.27	90.66 ± 12.19	89.94 ± 10.71	0.601
Twd (ms)	166.54 ± 33.87	175 ± 25.14	163.07 ± 31.19	166.6 ± 31.40	167.30 ± 31.70	0.768
QT (ms)	358.66 ± 25.29	364.4 ± 21.47	349.84 ± 27.6	363.40 ± 24.61	359.55 ± 24.98	0.359
QTc (ms)	393.28 ± 16.9	391.06 ± 22.35	385.53 ± 20.37	393.11 ± 16.11	392.04 ± 17.87	0.55
Tp-e (ms)	66.75 ± 13.14	69.33 ± 16.13	68.07 ± 11.09	70.18 ± 14.44	68.08 ± 13.57	0.73
Tp-e/QT	0.185 ± 0.034	0.189 ± 0.040	0.194 ± 0.030	0.19 ± 0.03	0.189 ± 0.035	0.753
Tp-e/QTc	0.169 ± 0.032	0.177 ± 0.041	0.176 ± 0.027	0.178 ± 0.035	0.173 ± 0.034	0.668
f(QRS-T) angle (degree)	26.63 ± 18.65	21.53 ± 21.02	22.46 ± 20.11	19.92 ± 16.82	23.84 ± 18.70	0.437

Data are presented as mean ± standard deviation for continuous variables and as number (percentage) for categorical variables. Group comparisons were performed using one-way ANOVA for continuous variables and Pearson's chi-square test for categorical variables. ASM, antiseizure medication; CBZ, carbamazepine; VPA, sodium valproate; LEV, levetiracetam; SBP, systolic blood pressure; DBP, diastolic blood pressure; BSA, body surface area; BMI, body mass index; HR, heart rate; Pdur, P-wave duration; PRint, PR interval; PRsegdur, PR segment duration; QRSdur, QRS duration; Twd, T-wave duration; QT, QT interval; QTc, corrected QT interval; Tp-e, Tpeak-Tend interval; Tp-e/QT, Tp-e to QT ratio; Tp-e/QTc, Tp-e to QTc ratio; F(QRS-T)angle, frontal QRS-T angle.

### 3.4 Echocardiographic parameters according to monotherapy ASM use

The comparison of the participants’ echocardiographic structural characteristics and diastolic and systolic function parameters and the antiepileptic drugs used in monotherapy were presented in Table 4. The LA-ML was significantly lower in the LEV group than in the control group (*P* = 0.041). The LAV was significantly higher in the CBZ, VPA, and LEV groups compared to the control group (*P* < 0.001, *P* < 0.001, and *P* < 0.001, respectively). Similarly, LAVi was also significantly higher in the CBZ, VPA, and LEV groups than in the control group (*P* < 0.001, *P* < 0.001, and *P* < 0.001, respectively). LAV was significantly higher in the CBZ group compared to the VPA and LEV groups (*P* = 0.036, *P* = 0.009, respectively). Likewise, LAVi was significantly higher in the CBZ group compared to the VPA and LEV groups (*P* = 0.033, *P* = 0.009, respectively). No significant differences were observed among the groups in terms of other echocardiographic parameters (*P* > 0.05).

**Table 4:** Comparison of echocardiographic systolic, diastolic, and left atrial measurements among ASM groups and control group.

Variables	Control (*n* = 57)	CBZ group (*n* = 15)	VPA group (*n* = 13)	LEV group (*n* = 27)	Total (*n* = 112)	*P*
EF (%)	63.12 ± 2.26	63.33 ± 2.25	61.46 ± 2.29	63.14 ± 2.46	62.96 ± 2.34	0.104
E/A	1.36 ± 0.40	1.43 ± 0.29	1.53 ± 0.69	1.44 ± 0.43	1.41 ± 0.44	0.645
E (cm/s)	95.81 ± 20.03	86.64 ± 9.45	100.67 ± 29.12	91.41 ± 20.73	94.08 ± 20.54	0.246
A (cm/s)	75.17 ± 20.37	61.86 ± 13.32	73.68 ± 22.16	66.70 ± 16.741	71.17 ± 19.3	0.055
E DT (ms)	160.43 ± 46.94	161.2 ± 37.23	188.53 ± 71.55	155.44 ± 29.84	162.59 ± 46.29	0.182
Lateral E’ (cm/s)	76.32 ± 19.18	70.90 ± 15.22	72.78 ± 15.09	69.59 ± 15.68	73.56 ± 17.49	0.369
IVRT (ms)	77.98 ± 12.11	81.06 ± 13.99	76.07 ± 9.81	77.96 ± 10.34	78.16 ± 11.65	0.718
FS (%)	46.23 ± 4.30	45.00 ± 6.08	44.56 ± 5.01	45.72 ± 5.10	45.75 ± 4.81	0.637
E/E'	9.23 ± 2.44	8.71 ± 2.77	8.19 ± 4.63	7.83 ± 2.62	8.70 ± 2.87	0.188
LA-AP (cm)	4.90 ± 0.44	4.84 ± 0.51	4.61 ± 0.41	4.95 ± 0.55	4.87 ± 0.48	0.19
LA-SI (cm)	3.84 ± 0.43	3.65 ± 0.53	3.73 ± 0.34	3.74 ± 0.50	3.78 ± 0.45	0.472
LA-ML (cm)	3.46 ± 0.35	3.56 ± 0.72	3.41 ± 0.49	3.17 ± 0.42	3.40 ± 0.46	0.024
LAV (ml)	40.91 ± 7.97	76.81 ± 15.24	62.63 ± 20.80	62.88 ± 16.63	53.53 ± 18.94	<0.001
LAVi (ml/m^2^)	23.63 ± 4.65	44.39 ± 8.81	36.13 ± 11.90	36.29 ± 9.60	30.92 ± 10.93	<0.001
LVEDD (cm)	4.41 ± 0.27	4.46 ± 0.31	4.39 ± 0.19	4.3 ± 0.23	4.39 ± 0.26	0.182
LVESD (cm)	2.36 ± 0.21	2.45 ± 0.39	2.42 ± 0.23	2.32 ± 0.18	2.37 ± 0.24	0.341
LVPWT (cm)	0.97 ± 0.13	0.93 ± 0.10	0.92 ± 0.11	0.89 ± 0.11	0.94 ± 0.12	0.067
IVS (cm)	0.94 ± 0.13	0.93 ± 0.11	0.93 ± 0.12	0.91 ± 0.12	0.93 ± 0.12	0.791
LVMI (g/m^2^)	79.92 ± 15.64	78.03 ± 17.38	73.93 ± 14.55	71.40 ± 11.8	76.92 ± 15.17	0.092
LVM (g)	141.85 ± 33.43	142.64 ± 34.24	135.08 ± 27.85	124.67 ± 26.70	137.03 ± 31.88	0.117
RWT	0.43 ± 0.06	0.41 ± 0.04	0.42 ± 0.05	0.42 ± 0.05	0.42 ± 0.05	0.676

All variables are presented as mean ± standard deviation. Group comparisons were conducted using one-way ANOVA. *post-hoc* analyses for pairwise comparisons were performed using the Tukey-B test. ASM, antiseizure medication; CBZ, carbamazepine; VPA, sodium valproate; LEV, levetiracetam; EF, ejection fraction; E, early mitral inflow velocity; A, late mitral inflow velocity; E/A, E to A ratio; E DT, E-wave deceleration time; Lateral E′, lateral mitral annular E′ velocity; IVRT, isovolumic relaxation time; FS, fractional shortening; E/E′, E to E′ ratio; LA-AP, left atrial anteroposterior diameter; LA-SI, left atrial superoinferior diameter; LA-ML, left atrial mediolateral diameter; LAV, left atrial volume; LAVi, left atrial volume index; LVEDD, left ventricular end-diastolic diameter; LVESD, left ventricular end-systolic diameter; LVPWT, left ventricular posterior wall thickness; IVS, interventricular septum thickness; LVMI, left ventricular mass index; LVM, left ventricular mass; RWT, relative wall thickness.

### 3.5 Association of treatment duration With LAVi in monotherapy ASM groups

**Figure 1:**
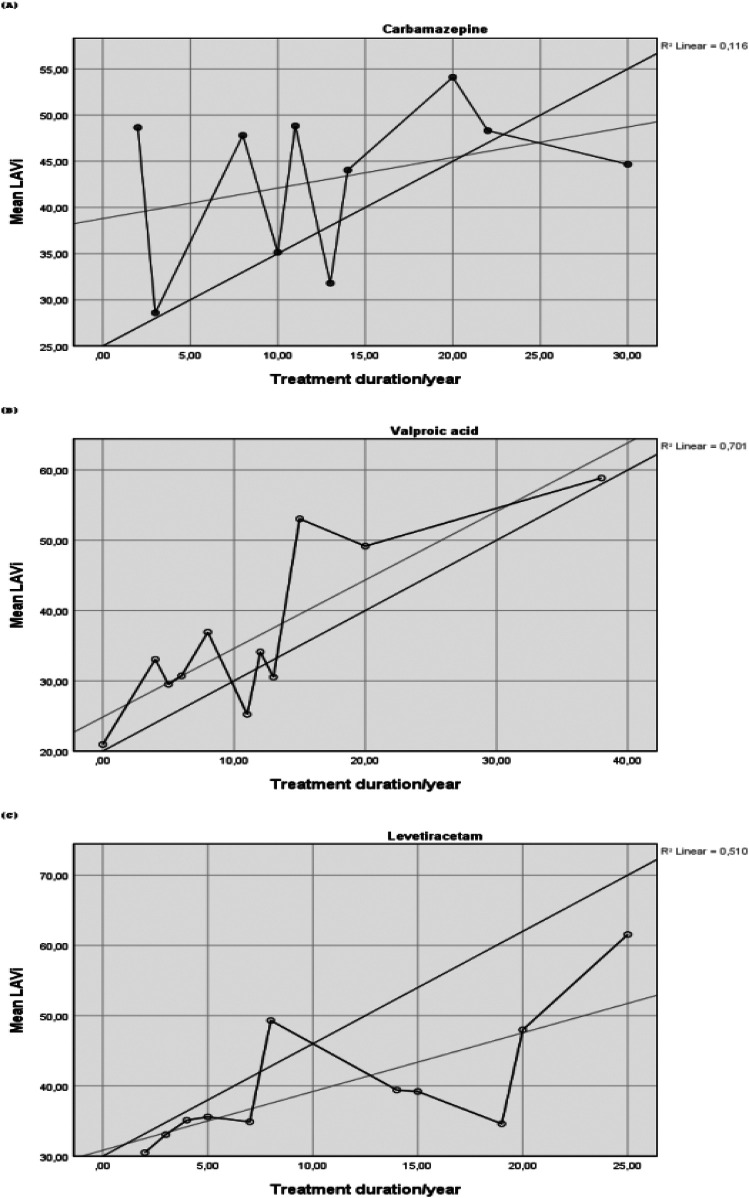
Correlation graphs showing the relationship between duration of ASMs and LAVi. **(A)** CBZ on top, **(B)** VPA in the middle, **(C)** LEV at the bottom.

The correlation between antiepileptic treatment duration and LAVi was shown in Table 5. No statistically significant correlation was observed between CBZ treatment duration and LAVi (r = 0.281, *P* = 0.311). A strong positive correlation was found between VPA treatment duration and LAVi (r = 0.776, *P* = 0.002). Additionally, a moderate positive correlation was observed between LEV treatment duration and LAVi (r = 0.571, *P* = 0.002). The correlation graphs illustrating the relationship between ASMs treatment duration and LAVi are presented in Figure 1.

**Table 5:** Correlation between treatment duration and LAVi in different ASM groups.

Treatment duration (years)	LAVi (ml/m^2^)	
	r	*P-*value
CBZ	0.281	0.311
VPA	0.776	0.002
LEV	0.571	0.002

Pearson correlation analysis. ASM, antiseizure medication; CBZ, carbamazepine; VPA, sodium valproate; LEV, levetiracetam; LAVi, left atrial volume index.

## 4 Discussion

In this study, no statistically significant difference was observed in ECG parameters, including repolarization and conduction indices, in drug-sensitive and drug-resistant epilepsy patients receiving different monotherapy regimens compared to healthy individuals. We observed that patients with epilepsy receiving ASM therapy exhibited significantly increased LAV and LAVi compared to the healthy control group. Furthermore, among ASM users, patients receiving CBZ demonstrated the highest LAV and LAVi values, significantly exceeding those of patients treated with VPA, LEV and the control group. These findings suggest a potential association between ASM use—particularly CBZ—and alterations in LA structure.

The present study demonstrates that epilepsy is associated with cardiac structural and functional alterations. In particular, studies have reported LA remodeling in people with epilepsy, with reduced LA emptying fraction and increased LAV/LAVi serving as early markers (13); these observations are directionally consistent with our finding of larger LA size, and extend prior syntheses documenting echocardiographic abnormalities in this population.

Although no prior study has directly demonstrated a link between enzyme-inducing ASMs and increased LAVI, recent work has shown LA remodeling with elevated LAVI in epilepsy patients. Within this context, our finding of CBZ-related LAVI enlargement is clinically relevant, as it provides hypothesis-generating evidence that may inform future evaluation of cardiac risk and monitoring strategies in epilepsy management.

The enlargement of the LA has been widely recognized as an early marker of diastolic dysfunction and an indicator of increased left ventricular filling pressure (14). CBZ, a sodium channel blocker, is known to induce cytochrome P450 (CYP) enzyme activity, which may contribute to reduced nitric oxide (NO) bioavailability through increased oxidative stress. This mechanism can accelerate the metabolism of vasodilatory substances and endogenous steroids, such as prostacyclin, potentially impairing vascular function (15, 16). This enzyme induction may contribute to chronic vascular remodeling, increased systemic vascular resistance, and subsequent left ventricular diastolic dysfunction, all of which can promote LA dilation. Moreover, CBZ has been reported to alter heart rate variability (HRV) and reduce parasympathetic tone, shifting the autonomic balance toward a more sympathetic-dominant state (17). This increased sympathetic activation may lead to chronic subclinical cardiac stress, promoting LA stretch and remodeling over time. Our findings align with prior research suggesting that CBZ therapy may be associated with altered cardiac structure, although direct echocardiographic evidence of LA enlargement in CBZ users has been limited until now.

In contrast to CBZ, VPA and LEV were associated with significantly lower LAV and LAVi values, closer to those observed in the healthy control group. VPA has been suggested to have cardioprotective effects due to its role in modulating GABAergic transmission, anti-inflammatory pathways, and oxidative stress reduction (18, 19). LEV, a non-enzyme-inducing ASM, has been shown to have minimal direct cardiovascular effects and is generally considered to have a more favorable cardiac profile compared to CBZ and VPA (7). In our study, patients taking LEV had the lowest LAV and LAVi values among ASM users, suggesting that LEV did not significantly contribute to LA remodeling and may be a preferred option in patients at risk of cardiac dysfunction.

Although the highest LAVi values were observed in the CBZ group, no statistically significant correlation was found between treatment duration and LAVi in this subgroup. This finding indicated that the observed increase in LAVi in the CBZ group was unlikely to be driven by treatment duration, and may instead reflect drug-specific effects occurring independently of cumulative exposure. In contrast, a strong positive correlation between VPA treatment duration and LAVi was observed, indicating a potential time-dependent effect of this drug on atrial structure. While preclinical studies have suggested that VPA may reduce atrial remodeling and dilatation in experimental models (20), our findings indicated that, in clinical settings, long-term VPA use may be associated with increased LAV. The moderate correlation observed with LEV suggested a potential gradual influence on atrial dimensions, although the underlying mechanisms remain to be elucidated. These findings emphasized the importance of evaluating both drug-specific and time-dependent effects when assessing cardiac structural changes in patients with epilepsy.

The ECG abnormalities, particularly those related to repolarization indices such as QT, QTc, Tp-e interval and f(QRS-T) angle have been widely studied in epilepsy due to concerns regarding SUDEP (21, 22). Evidence on ventricular repolarization is mixed. Some reports and reviews emphasize QT/QTc abnormalities and their relevance to SUDEP risk (23, 24), whereas others found no consistent association between specific ASMs and QTc when methodological differences in QTc calculation are accounted for (25). Prior studies have suggested that certain epilepsy syndromes and ASMs can modulate autonomic tone and cardiac electrophysiology, potentially leading to pro-arrhythmic conditions (26). In our study, no significant differences in ECG parameters were observed between the drug-sensitive, drug-resistant, and control groups. The lack of significant ECG changes in our study population could be due to the collection of patients in the interictal period of epilepsy. Additionally, it has been suggested that autonomic dysfunction in epilepsy patients may not always have significant ECG abnormalities in short-term evaluations, but may be more evident during autonomic stress tests, Holter monitoring, or tilt-table testing (27, 28).

The clinical relevance of LA enlargement in epilepsy patients was an important consideration. Increased LAV and LAVi have been associated with higher risks AF, stroke, and heart failure in the general population (29–31). Given the elevated risk of SUDEP and cardiovascular comorbidities in epilepsy patients, routine cardiac screening using TTE may be warranted, particularly in those receiving enzyme-inducing ASMs like CBZ. Future studies should evaluate whether long-term CBZ therapy contributes to progressive LA enlargement and subsequent cardiac complications.

The implications of our findings for clinical practice should be interpreted with caution. Although our results suggest that ASM therapy—particularly with enzyme-inducing agents—may contribute to LA remodeling, repolarization variability indices were not significantly altered. This indicates that structural rather than electrophysiological changes may be more consistently detectable in this population. From a practical perspective, our findings do not directly modify existing management strategies, but they highlight the potential importance of cardiac monitoring in epilepsy patients receiving long-term ASM therapy.

This study is among the first to examine both structural and electrophysiological cardiac changes in epilepsy within the same cohort, using echocardiographic and ECG measures in parallel. Our results contribute to the literature by showing that ASM exposure, particularly to enzyme-inducing agents, may be associated with LA enlargement, while repolarization variability indices were not significantly altered. These findings underscore the value of multimodal cardiac assessment in epilepsy research and provide hypothesis-generating data for prospective validation.

### 4.1 Limitations

Despite its strengths, our study had some limitations. First, longitudinal follow-up data were not available, making it difficult to determine whether the observed LA enlargement is progressive over time. Second, we did not assess cardiac biomarkers like brain natriuretic peptide and troponin, which could have provided additional insights into myocardial strain. Third, the lack of untreated epilepsy patients limits the ability to clearly differentiate the cardiac effects of epilepsy from those induced by ASMs. Fourth, our study included only three ASMs—CBZ, VPA and LEV. However, the exclusion of newer third and fourth generation ASMs limited the generalizability of our findings. Finally, although we accounted for confounding factors such as HT and BMI, other potential contributors (e.g., sleep apnea, seizure frequency) were not fully explored.

### 4.2 Future directions

Future research should include long-term, prospective, and multicenter studies to evaluate whether CBZ-induced LA enlargement is reversible upon ASM withdrawal or substitution. In addition, future investigations should incorporate cardiac biomarkers and comprehensive assessments of autonomic function, seizure burden, and comorbid conditions to better clarify the mechanisms underlying ASM-related cardiac changes. Comparative studies involving newer-generation ASMs may also help determine whether the observed effects are drug-specific or class-related, ultimately guiding clinical monitoring strategies in epilepsy management.

## 5 Conclusion

Our study demonstrated that epilepsy patients receiving ASM therapy exhibited significant LA enlargement, with the greatest increase in LAV and LAVi observed in the CBZ group. LAVi showed a significant positive correlation with treatment duration in the VPA and LEV groups, whereas no such association was found in the CBZ group. This pattern suggests that the LA enlargement observed with CBZ may be drug-specific and independent of cumulative exposure. Repolarization indices did not differ significantly among the groups; however, this finding suggests that future studies should evaluate the role of serial or prolonged ECG monitoring in epilepsy patients rather than relying solely on a single baseline recording. Overall, our data underscore the need for prospective, multicenter studies to further clarify the long-term cardiovascular risks associated with ASM-related cardiac changes and to guide future clinical practice.

## Data Availability

The raw data supporting the conclusions of this article will be made available by the authors, without undue reservation.
